# Electronic optional guidelines as a tool to improve the process of referring patients to specialized care: An intervention study

**DOI:** 10.3109/02813432.2013.824155

**Published:** 2013-09

**Authors:** Ingrid S. Rokstad, Kirsten S. Rokstad, Sissel Holmen, Sverre Lehmann, Jörg Assmus

**Affiliations:** ^1^Haukeland University Hospital, Norway; ^2^Department of Research and Development, Haukeland University Hospital; ^3^Department of Thoracic Medicine, Haukeland University Hospital; ^4^Centre for Clinical Research, Haukeland University Hospital

**Keywords:** General practice, general practitioners, intervention studies, lung diseases, Norway, outpatients, quality improvement, referral and consultation

## Abstract

**Objective:**

The main objective of this paper is to investigate whether incorporating an electronic optional guideline tool (EOGT) in the standardized referral template used by general practitioners (GPs) when referring patients to specialized care can improve outpatient referral appropriateness.

**Design:**

Intervention study with an intervention and a control group.

**Setting:**

210 GPs in the municipality of Bergen and the Department of Thoracic Medicine at Haukeland University Hospital.

**Subjects:**

2400 patients referred to the Department of Thoracic Medicine at Haukeland University Hospital.

**Results:**

An electronic optional guideline tool (EOGT) was implemented on 93 of 210 GPs’ computer systems. The referral quality and the time spent reviewing each referral were evaluated by the hospital specialists. The GPs did not know that their referrals were being evaluated. The specialists were blinded with regard to information concerning the intervention and the control group. The specialists reported significantly higher referral quality and considerably less time spent on evaluating referrals when using the EOGT, with an overall time reduction of 34%. Likewise, GPs also reported that the EOGT was easy to use, time-saving and led to an improved quality of their referrals.

**Conclusion:**

This study documents an improvement in the quality of the referrals. Since the GPs save time by using the EOGT, there is no reason to believe that they will discontinue using it. In fact, the tool may be even more beneficial for the GP. The authors believe that it is possible to implement the EOGT as a standard referral tool within various fields of medicine and are currently in the process of developing these tools.

- The specialists reported significantly higher referral quality and considerably less time spent on evaluating referrals written by GPs using the electronic optional guideline tool (EOGT).
- The overall time reduction using the EOGT was 34%.
- The GPs reported that the EOGT was easy to use, time-saving, and led to improved quality of their referrals.

## Introduction

Every year Norwegian GPs spend 150 work years writing approximately 1.9 million referrals to specialists [1]. The quality of these referrals is important both for the appropriate care of the patients and for the economics of the health-care system [2]. However, the number of work years consumed by specialists in processing these referrals is not known.

Recent studies have shown that information communicated through referrals is often insufficient in spite of their far-reaching implications and the importance of their being appropriate [3,4]. This means that the process of managing referrals between GPs and specialists is in need of improvement to secure the interests of the patients and relieve specialists of the extra time spent on managing poor referrals [5,6]. Previous intervention studies on the quality of GP referrals have shown improvements [7,8] but have indicated that it is difficult to maintain the results over time [7,8] . The aim of this study was to investigate whether incorporating an electronic optional guideline tool (EOGT) in the standardized referral template used by GPs when referring patients to specialized care could improve outpatient referral appropriateness and maintain this improvement over time.

## Materials and method

In this study one GP and three pulmonary specialists at the Department of Thoracic Medicine at Haukeland University Hospital have collaborated to improve the quality of referrals from GPs to the specialists at the hospital. Collectively they have compiled a set of indicators for three different diagnoses, namely sleep apnoea (SA), chronic obstructive pulmonary disease (COPD), and tumours of the lung (LT). These indicators represent information that the pulmonary specialists consider to be most important regarding the review of referrals from GPs. The GPs also considered that using these indicators was feasible in general practice. Based on these indicators, the EOGT was created in order to increase the effectiveness and efficiency of the referral process.

**Figure 1.**
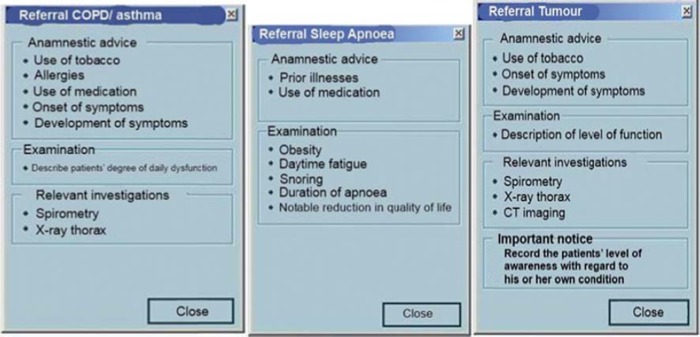
The information box above appeared in the upper right corner of the electronic standardized referral template when the GP selected COPD, SA, or LT as the reason for referral.

The outpatient clinic at the Department of Thoracic Medicine, Haukeland University Hospital, annually receives about 2400 patient referrals. Of these, 60% are diagnosed with SA, COPD, or LT. For each of the diagnoses seven or eight indicators were listed. When the GPs wrote a referral to a specialist using this tool, the GPs first decided on a tentative diagnosis using clinical skills and knowledge. If this tentative diagnosis was an LT, SA, or COPD, the EOGT provided an information box (Figure 1) corresponding to the diagnosis at hand. This provided a reminder regarding which information the GP should provide in order to increase the appropriateness of the referral. In Norway it is common practice for most GPs to write the referral while the patient is present in the consultation room. This means that the GP can use the EOGT guidelines to gather information about the patient directly and simultaneously write the referral.

The EOGT was implemented in Infodoc Plenario, a computer system currently used by 93 GPs in the municipality of Bergen (n = 93). This took place in conjunction with a new version of the software being installed. This version included many improvements in addition to the EOGT. The remainder of the GPs (n = 117) who were not using this particular computer system were included in a control group. The GPs were not informed that their referrals would be subject to anonymous evaluation and comparison with a control group in this study. During the intervention period the same evaluation form was used to evaluate all referrals sent from these GPs to the Department of Thoracic Medicine, Haukeland University Hospital. This was performed over a period of nine months by six specialists in pulmonary medicine. They were all consultants working full time. The specialists were blinded from all information regarding the intervention and control groups. The amount of time the specialist used to consider and manage each referral was recorded using a stopwatch. They were instructed to record 30 to 60 seconds as one minute and less than 30 seconds as zero.

The evaluation forms contained two identical questions regarding referrals of all three diagnoses, namely: “Does the referral communicate a clear message?” and “Is the referral overall easily read?” In addition, there were seven other questions regarding referrals for SA and eight questions regarding LT and COPD. The specialists were instructed to score the referrals/diagnoses on a scale from zero to 12, 12 being the optimal score. The total score for each referral was calculated thereafter.

After the evaluation the GP intervention group was also interviewed by telephone regarding whether they used the new tool (yes/no), whether they found it easy to use (yes/no/indifferent) and whether their time expenditure on writing referrals after the electronic tool was implemented had changed (increased/decreased/unchanged). The GPs were also asked whether they felt that their referrals were more appropriate, the same, or less appropriate than before using the new tool.

### Statistical method

Descriptive statistical methods were used to analyse the data collected during this study. Tests between the two continuous variables “time” and “total score” were carried out using the Wilcoxon test. The chi-square or Fisher's exact test was applied when comparing categorical variables. The significance level was 0.05. To account for multiple effects, the significance level was adjusted using the Bonferroni correction, thus leading to an adjusted level of 0.0071 for SA (seven tests) and 0.00625 for COPD and LT (eight tests).

## Results

Every GP in the municipality of Bergen was included in this study (n = 210). Of these, 93 constituted the intervention group. There were no differences between the two groups with regard to main variables such as gender, age, and whether or not they were specialists in general practice. During the nine months in which the study took place, a total of 1080 new referrals for the three diagnostic groups SA, COPD, and LT were received by the hospital. Of these, 664 referrals (70%) were from GPs. A small number of referrals may have come from GPs in districts not included in the study. These, however, do not use the Infodoc Plenario system and can therefore be considered as part of the control group.

**Figure 2.**
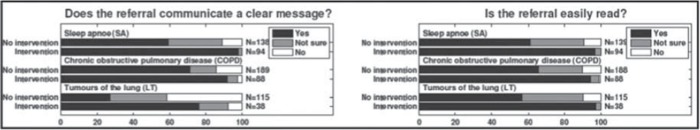
This figure shows the two main questions in common for all three diagnoses and that the use of these two questions results in a significant improvement in the most important part of a referral: Does the referral communicate a clear message and is the referral easily read.

The evaluation scores in this study show an average overall improvement of almost 30% for all three diagnostic groups. The two questions in common for evaluating all referrals showed significant differences between the intervention and control group (Figure 2).

**Figure 3.**
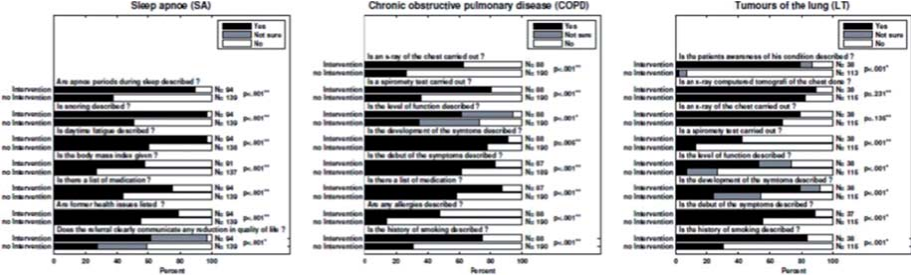
Percentage of the answers to questions 3–10 for sleep apnoea, chronic obstructive pulmonary disease, and lung tumours in the intervention and the non-intervention group in numbers and percentages with p-values of the chi-squared test (*) or Fisher's exact test (**).

Two of the parameters regarding lung tumours showed no significant differences, namely chest X-ray (p = 0.142) and CT thorax (p = 0.234) (Figure 3). All other parameters showed significant differences (Figure 3).

There were no signs of burnout tendencies on the degree of intervention effect when comparing the results from the first two with the last two months when analysing the two questions in common for evaluating all referrals (see Figure 2). These two groups were used to compare the degree of burnout since these groups were large enough to be statistically significant. All (the) questions with the exception of two showed this tendency; however, some numbers were too small to use in the statistical calculation. The average total amount of time spent by the specialists on evaluating referrals from GPs in the intervention group was 34% shorter than in the control group (SA 42%, COPD 23%, and LT 41%); 82 of the GPs had used the EOGT. The 11 GPs not using the tool reported that they had not been aware that the tool was implemented in the new version of their computer software. Of the 82 GPs who reported using the tool, 96% found it saved time, and all found it easy to use and were under the impression that they recorded better information for the specialists to review.

## Discussion

One weakness of the study could be that the GPs in Bergen were not randomized into an intervention and a control group, making system bias a possible weakness. The GPs using Infodoc Plenario received the EOGT as part of a new version of the software containing other additional improvements. One could argue that the GPs who had installed the newest version of the Infodoc software system are more attentive and may have written better referrals with or without the EOGT. The analysis of the intervention and control group showed no differences in whether the GP was a specialist or not, which strengthens our belief that this systematic bias does not occur. Furthermore, neither the intervention group nor the control group knew that their referrals were subject to anonymous evaluation; 88% of the GPs in the intervention group used the EOGT at all times. The referrals made by 12% of the GPs will not cause any methodological problem because it will only reduce the differences between the two groups.

We believe one weakness of the study is that some referrals were included from GPs outside Bergen. However, none of these was written using the EOGT as these GPs do not use the Infodoc Plenario software. This may explain why 44% of the GPs in the intervention group wrote only 33% of the referrals. We also have to take into consideration that 11 of the GPs included in the intervention group did not use the EOGT.

The GPs were interviewed after the evaluation was completed; however, they were still using the EOGT. At the time of the interview the GPs had become more comfortable using the tool and this may have positively influenced the answers given in the interview.

A meta-analysis showed that interventions involving advice obtained from a structural referral sheet were the only effective measure for improving a referral [9]. Other studies have shown the same results [6,7]. Our study supports this finding, as only two parameters were unchanged, chest X-ray and CT scan of lung tumours, which not unexpectedly had a high score both in the control and in the intervention group. Several intervention studies show that the effect of an intervention diminishes over time if the intervention is not repeated regularly [7,8]. The greatest benefit of the EOGT is that it serves as an ongoing intervention because it appears every time the GP uses the structured referral template. Thus, its effect is more likely to be withheld. Comparing the first with the last months of the study we found no tendency towards burnout.

Grimshaw et al. pointed out that referral guidelines are more likely to be effective if local secondary care providers are involved with GPs in making guidelines [9]. It is important to both reflect local circumstances and address local barriers. This survey was a collaborative project between well-respected, local pulmonary specialists at the hospital and an experienced GP. This may also contribute to explaining the great improvement in the intervention group.

It has been shown that the main disadvantage of structural referral templates is that more time is required to fill them out and that their effect diminishes with time [7,9]. To meet this challenge, this study has attempted to provide the GPs with a tool that is easy to use and contains structural advice from specialists, which reduces the workload, saves the GPs time, and helps them write referrals of better quality. The scores in this study show an average overall improvement of almost 30% for all three diagnosis groups. This corresponds with another study that assesses quality improvement of referrals from primary health care [10]. Ranvolt et al. found a 36% increase in adequate referrals using an intervention composed of simple but precise guidelines for referrals in paper format. Their results were based on a registration period of only three months compared with our study that lasted nine months [10]. The Danish study also showed that more than 70% of referrals to gynaecologic and orthopaedic departments did not contain any information on the patients’ use of medicines [10]. Our study showed that no information on the patients’ use of medicines in the control group was provided in 44% of the referrals for sleep apnoea and 58% for referrals regarding COPD (see Figure 3). Using the optional guidelines there was a significant increase in both groups (SA and COPD) after the intervention of 44% to 76% and 58% to 87%, respectively.

Kristoffersen et al. show that in order to succeed in implementing an EOGT, the tool has to function well in day-to-day general practice and be easy to use [12]. Heimly tried to implement an interactive EOGT, but concluded that this was too time-consuming for the GPs [13].

The negative outcome of implementing referral guidelines in general practice developed by hospital specialists alone has been discussed by Jiwa et al. [11]. They pointed out that specialists were too demanding and that it was too time-consuming for the GPs to collaborate. In our study we have shown that not only the specialists but also the GPs saved time using the guidelines. After using the EOGT for one year the GPs in Bergen forwarded a request that Haukeland University Hospital take charge of developing EOGTs for other fields of medicine. This is currently taking place.

## Conclusion

Time is limited in outpatient clinics today and the demand for efficiency is therefore high. Hence, new methods are necessary to improve efficiency and quality at the outpatient clinics. Our study documents an improvement in the quality of the referrals. Since the GPs save time using the EOGT and report that they find it very useful, there is no reason to believe that they will discontinue using it. The EOGT benefits all parties, in particular the GP. Therefore it is logical to assume that the intervention tool will continue to have a time-saving and quality-improving effect on the referrals. We believe that it is also possible to implement the EOGT as a standard referral tool within various other fields of medicine on a national basis for all GP software systems. Haukeland University Hospital is currently developing such tools for investigating urological and gastroenterological cancer diagnoses.
